# Full Tensor Eigenvector Analysis on Air-Borne Magnetic Gradiometer Data for the Detection of Dipole-Like Magnetic Sources

**DOI:** 10.3390/s17091976

**Published:** 2017-08-29

**Authors:** Boxin Zuo, Lizhe Wang, Weitao Chen

**Affiliations:** School of Computer Science, China University of Geosciences, Wuhan 430074, China; boxzuo@163.com (B.Z.); wtchen@cug.edu.cn (W.C.)

**Keywords:** magnetic gradiometer, eigenvector analysis, magnetic dipole, unexploded ordnance detection

## Abstract

The detection of dipole-like sources, such as unexploded ordnances (UXO) and other metallic objects, based on a magnetic gradiometer system, has been increasingly applied in recent years. In this paper, a novel dipole-like source detection algorithm, based on eigenvector analysis with magnetic gradient tensor data interpretation is presented. Firstly, the theoretical basis of the eigenvector decomposition of magnetic gradient tensor is analyzed. Then, a detection algorithm is proposed by using the properties of the tensor eigenvector decomposition to locate dipole-like magnetic sources. The algorithm can automatically detect magnetic dipole-like sources without estimating the magnetic moment direction. It performs well for locating weak, anomalous dipole-like sources in air-borne magnetic data through quantitative interpretation. The effectiveness of the proposed algorithm has been demonstrated in the designed synthetic experiment. Finally, an air-borne magnetic field data taken at high altitude with exact source position information is used to validate the practicality of the proposed algorithm. All of the experiments prove that the proposed algorithm is suitable for magnetic dipole-like source detecting and air-borne magnetic gradiometer data interpretation.

## 1. Introduction

An estimated million acres of land throughout the world are contaminated with various types of unexploded ordnances (UXO), landmines, and other steel objects [1]. To clean up these areas, magnetic surveys are one of the most common geophysical methods used to detect these metallic targets [2]. However, discriminating these magnetic sources with magnetic field data is a challenging task. To characterize and locate potential UXO targets precisely, many methods for automatic detection, localization, and classification (DLC), using total magnetic intensity (TMI) data, have been developed [2,3]. Kristofer et al. proposed using an extended Euler deconvolution algorithm for automatic UXO detection based on the concept of the structural index (SI) [4]. Zalevsky et al. defined a wavelet mother function with the physical parameters of a magnetic dipole for detecting marine field magnetic targets [5]. Phillips et al. suggested using the Helbig method to estimate magnetization direction before applying the Euler analysis to locate compact magnetic sources [6]. Li et al. formulated a downward continuation algorithm as an inverse problem with Tikhonov regularization for enhancing magnetic data acquired for UXO discrimination [7].

Recent advances in magnetic gradiometry have significantly increased the signal to noise ratio (SNR) of magnetic gradient tensor (MGT) data. Magnetic gradiometry has been increasingly used in wide area assessments to rapidly locate potential UXO contamination [8]. Schneider et al. utilized the Wynn Frahm algorithm for inverse full-tensor data from dipole-like sources with a ground-based magnetic SQUID gradiometer system [9]. Sanchez et al. presented the numerical modelling of UXO and related metallic objects with a nonlinear integral equation [10]. Their results show that the presence of significantly higher order moments of asymmetric objects are obviously above the noise level of ground observed magnetic gradient data. Beiki et al. proposed the normalized source strength (NSS) method to deal with the remanent magnetization effect for quantitative interpretation of magnetic gradiometer data [11]. Yin et al. proposed modified tilt angles and using the NSS method to detect multiple dipole sources by using magnetic gradient tensor data [8]. Munschy et al. proposed a non-linear dipole inversion algorithm for ground magnetometer-measured profiles data in order to locate and characterize dipoles generated by UXO [12]. Hiergeist et al. built a ground level test facility to analyze signal resolution and the relative spatial resolution of MGT measured with unexploded bomb detection applications [13]. Bracken and Brown used a prototype tensor magnetic gradiometer system and designed a data-reduction procedure to detect UXO [14]. These advanced discrimination methods have been successfully deployed in UXO survey activities with ground-based magnetometry and magnetic gradiometry systems. 

In order to expand the magnetic gradiometry survey over large areas and over areas that are unsuitable for deploying ground-based systems, airborne magnetic gradiometry systems have been developed. Airborne magnetometer surveys can rapidly screen both surface and subsurface steel objects without being affected by the geological background noise. Billings and Wright deployed a low-level helicopter magnetometer survey and proved its ability to perform wide-area UXO assessment [15]. Doll et al. showed that the source of noise for low-altitude air-borne systems generally comes from the helicopter rotor [16]. Xia et al. suggested a moving hum filter to suppress the rotor noise in helicopter-based airborne systems for the detection of UXO [17]. Burazer et al. suggested using a FIR filter to extract weak anomalies from data that were measured at high-altitude air-borne magnetometer system [18]. As Billings and Wright analyzed, a large sensor and target stand-off distances of an airborne magnetometer system will result in a reduction in the amplitude anomalies [15]. Therefore, to process data obtained at high altitudes, it is necessary to extract the weak anomalous information caused by small buried steel objects. Miller and Singh suggested that when the observation distance is 2.5 times larger than the largest size of the source, the magnetic source can be considered as a magnetic dipole [19]. Therefore, UXO and other metallic targets in high-altitude air-borne magnetometer surveys can be considered to be dipole-like sources. 

In recent years, eigenvalue analysis has become one of the most popular methods applied to geophysical data processing. Fakiris et al. presented an automated target detection approach with Principal Component Analysis (PCA) for marine magnetic data interpretation [20]. Zou and Nehorai derived the analytical expressions of magnetic gradient tensor and presented an efficient ship wake detection method for airborne magnetic data [21]. Zuo and Hu proposed an eigenvalue analysis method to detect geological structures in air-borne gravity gradiometer data processing [22]. Beiki and Pedersen developed an eigenvector analysis method for air-borne gravity gradient tensor data to interpret geologic structures [23]. In this paper, we propose a high-order tensor eigenvector method to detect magnetic dipole-like sources in air-borne magnetic gradient tensor data. The reliability and efficiency of the proposed method is analyzed with a designed synthetic data experiment. Finally, the method is applied to the air-borne magnetic field data of the Fort Peck reservation, Montana, USA.

## 2. Methods

The magnetic gradients present the spatial rates of change of the magnetic field B (Vs/m^2^, SI Unit) in Equation (1) [24].

(1) $$B=-C_{m}\nabla \varphi =-C_{m}\nabla \int_{V}\nabla_{\left(r_{i}\right)}\frac{1}{\left|r-r_{i}\right|}\cdot m(r_{i})dv$$

where $m(r_{i})$ is the magnetization vector of the sources, **r** and **r**_i_ denote observation and source positions, respectively. $C_{m}=10^{-7}$ (Henry/m, SI units). Then, the magnetic gradient tensor **Γ** can be written as shown in Equation (2).

(2) $$\Gamma =-C_{m}\left[\begin{array}{ccc} \frac{\partial^{2}\varphi }{\partial x^{2}} & \frac{\partial^{2}\varphi }{\partial x\partial y} & \frac{\partial^{2}\varphi }{\partial x\partial z}\\ \frac{\partial^{2}\varphi }{\partial y\partial x} & \frac{\partial^{2}\varphi }{\partial y^{2}} & \frac{\partial^{2}\varphi }{\partial y\partial z}\\ \frac{\partial^{2}\varphi }{\partial z\partial x} & \frac{\partial^{2}\varphi }{\partial z\partial y} & \frac{\partial^{2}\varphi }{\partial z^{2}} \end{array}\right]=\left[\begin{array}{ccc} \Phi_{xx} & \Phi_{xy} & \Phi_{xz}\\ \Phi_{yx} & \Phi_{yy} & \Phi_{yz}\\ \Phi_{zx} & \Phi_{zy} & \Phi_{zz} \end{array}\right]$$

where $\Phi_{ij}(i=x,y,z;j=x,y,z;)$ denotes the elements of the magnetic gradient tensor. Assuming the magnetic gradient tensor matrix **Γ** can be diagonalized as $\Lambda =V^{\prime }\Gamma V$, and eigenvector V($V=\left[V_{1},V_{2},V_{3}\right]$) forms a new coordinate system. Physically, this diagonalized procedure rotates the coordinate system to align with the equivalent point source direction [25]. **Γ** can be projected to a new form coordinate system with the diagonalized procedure. The rotation matrix can be expressed as Equation (3):

(3) $$V=\left[\begin{array}{ccc} \cos\psi \cos\theta \cos\phi -\sin\psi \sin\phi & \cos\psi \cos\theta \cos\phi -\sin\psi \sin\phi & \cos\psi \sin\theta \\ -\sin\psi \cos\theta \cos\phi -\cos\psi \sin\phi & -\sin\psi \cos\theta \sin\phi -\cos\psi \cos\phi & -\sin\psi \cos\phi \\ -\sin\theta \cos\phi & -\sin\theta \cos\phi & \cos\theta \end{array}\right]$$

where ψ,θ,ϕ are the rotation angles about *x*-, *y*-, and *z*-axis, respectively. After decomposition, the non-diagonal elements are much smaller than the diagonal elements and the magnetic dipole direction information is extracted to the rotation matrix V. Hasan et al. suggested a method to calculate the matrix V through the eigenvector decomposition for 3-D tensor data as Equation (4) shows:

(4) $$v_{i}=\left[\begin{array}{c} \left[\Phi_{xy}\Phi_{yz}-\Phi_{xz}(\Phi_{yy}-\lambda_{i})\right]\left[\Phi_{xz}\Phi_{yz}-\Phi_{xy}(\Phi_{zz}-\lambda_{i})\right]\\ \left[\Phi_{xz}\Phi_{yz}-\Phi_{xy}(\Phi_{zz}-\lambda_{i})\right]\left[\Phi_{xz}\Phi_{xy}-\Phi_{yz}(\Phi_{xx}-\lambda_{i})\right]\\ \left[\Phi_{xy}\Phi_{yz}-\Phi_{xz}(\Phi_{yy}-\lambda_{i})\right]\left[\Phi_{xz}\Phi_{xy}-\Phi_{yz}(\Phi_{xx}-\lambda_{i})\right] \end{array}\right]$$

where the largest eigenvalue $\lambda_{i}$ can be estimated with the tensor components $\Phi_{ij}$ [26]. Li suggested that this diagonalized decomposition rotates the local coordinate system into a user-defined system, and the Euler angle of the rotated coordinate is $\theta^{\prime }=0,\;\psi^{\prime }=0$ [25]. 

In this paper, we propose a magnetic dipole-like location method using eigenvector analysis. According to Equation (3), the eigenvector rotates angle ϕ along the *z*-axis after eigenvector decomposition. If the angle ϕ is not equal to zero, it means that the observation point has a vertical direction bias when compared to the position of the magnetic dipole source, while the magnetic dipole moment will be represented by the corresponding eigenvalue. From this view, we think that the purpose of the eigenvector analysis is to decompose the magnetic gradient data into two data sets: the eigenvector data set contains the source position information, and the magnetic moment is represented by the eigenvalues data set. Through this procedure, noise and interference error can be suppressed because they do not have an anomalous response in all five independent tensors ($\Phi_{xx}$, $\Phi_{xy}$, $\Phi_{xz}$, $\Phi_{yy}$, $\Phi_{yz}$).

**Figure 1:**
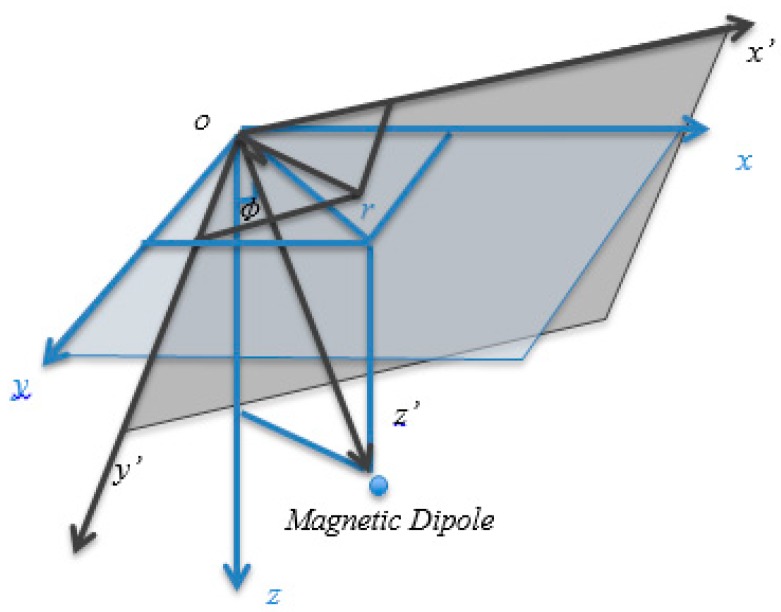
Coordination rotation by eigenvector $V_{1}$ decomposition; Angle ϕ is the coordination rotation angle.

According to this deduction, it is possible to locate magnetic dipole-like sources by using tensor eigenvector decomposition. To make the value of the eigenvector inversely proportional to the position of the magnetic dipole, the direction of eigenvector $v_{1}=[x^{\prime },y^{\prime },z^{\prime }]$ is defined through the angle ϕ, as shown in Figure 1.

Theoretically, the eigenvector $v_{1}$ is a unit vector. The amplitude will not commonly equal 1 with the noise interference. But the error of amplitude will not influence the direction of eigenvector $v_{1}$. The angle ϕ can be calculated with Equation (5).

(5) $$\mathrm{ctan}\phi =\frac{\left(\Phi_{xy}\Phi_{yz}-\Phi_{xz}(\Phi_{yy}-\lambda_{1})\right)\left(\Phi_{xz}\Phi_{xy}-\Phi_{yz}(\Phi_{xx}-\lambda_{1})\right)}{\left(\Phi_{xz}\Phi_{yz}-\Phi_{xy}(\Phi_{zz}-\lambda_{1})\right)\left(\left(\Phi_{xy}\Phi_{yz}-\Phi_{xz}(\Phi_{yy}-\lambda_{1}))+(\Phi_{xz}\Phi_{xy}-\Phi_{yz}(\Phi_{xx}-\lambda_{1})\right)\right)}$$

A small angle ϕ rotation will result in a relative large ctanϕ value to illustrate that the position of the dipole is close to the observation point. Conversely, if the value of ctanϕ is nearly equal to 0, it indicates that there is no source nearby to this observation position. Through this transformation, the position of the source is projected to a source position map, and it is then able to be visually analyzed. Here, we define the Magnetic Dipole Tangent Angle (MDTA) according to the rotation angle ϕ, as expressed in Equation (6).

(6) $$\mathrm{MDTA}={\left\lceil \mathrm{ctan}\phi \right\rceil }_{\alpha }$$

Although, theoretically, the noise in full tensor data will not present a local peak value in MDTA, a high-level noise and low quality data will create false alarms in the MDTA map in practice. Therefore, a threshold filter ${\left\lceil \cdot \right\rceil }_{\alpha }$ was set to remove these small false alarms, which are much smaller than the peak value that is caused by a source.

## 3. Method Validation

In this section, three synthetic experiments were designed to validate the proposed algorithm. The performance of the proposed algorithm with different dipole moment directions and multiple-source scenarios are discussed in detail.

**Figure 2:**
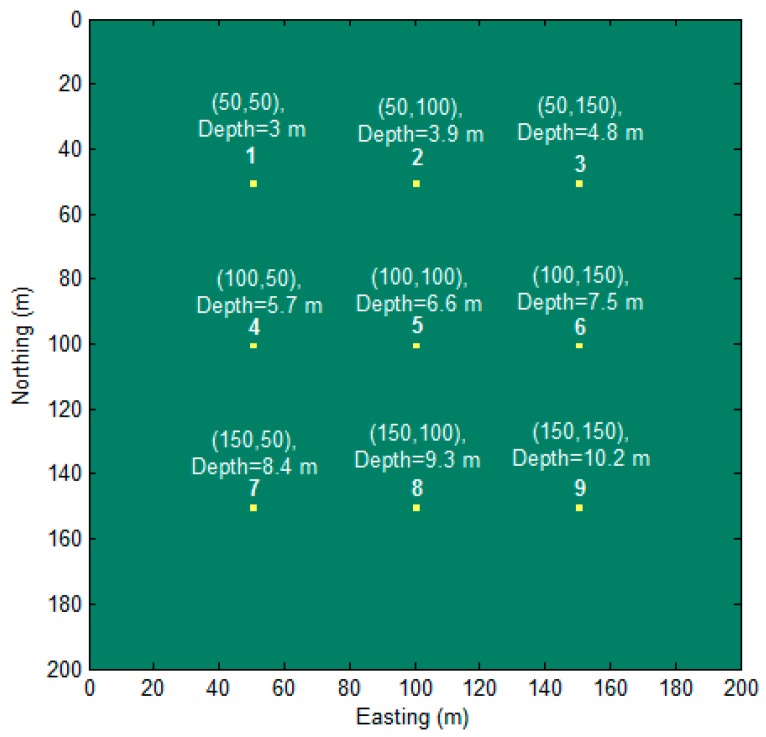
Plain sectional view of the synthetic model.

Firstly, a synthetic model was designed with nine magnetic dipoles that are buried in different depths, as shown in Figure 2.

**Figure 3:**
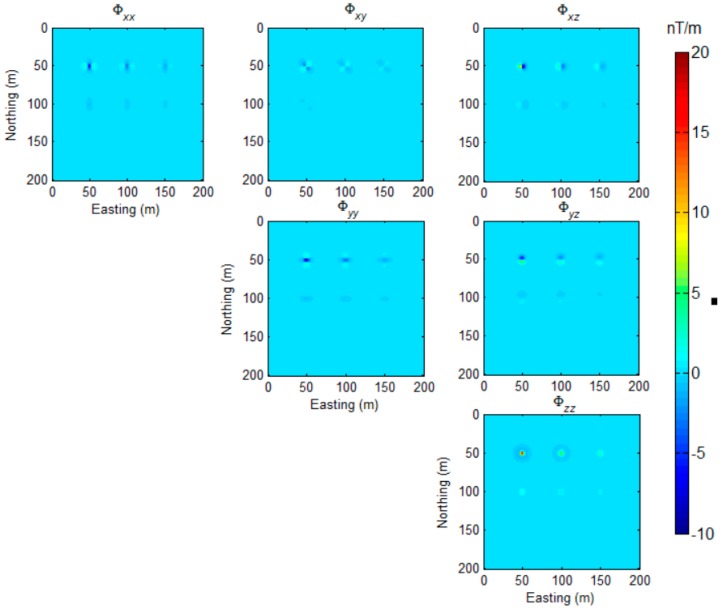
The simulated magnetic gradient tensor data.

The moment of the dipoles was 20 Am^2^, and the declination and inclination of the dipoles magnetization vector were set as $I=90^{\circ }，D=0^{\circ }$. The simulated magnetic field was sampled at an interval of 1 m in the horizontal plane. The full magnetic gradient components of the magnetic field were calculated according to the equations that were proposed by Pedersen and Rasmussen [27]. These magnetic gradient tensors are displayed in Figure 3.

**Figure 4:**
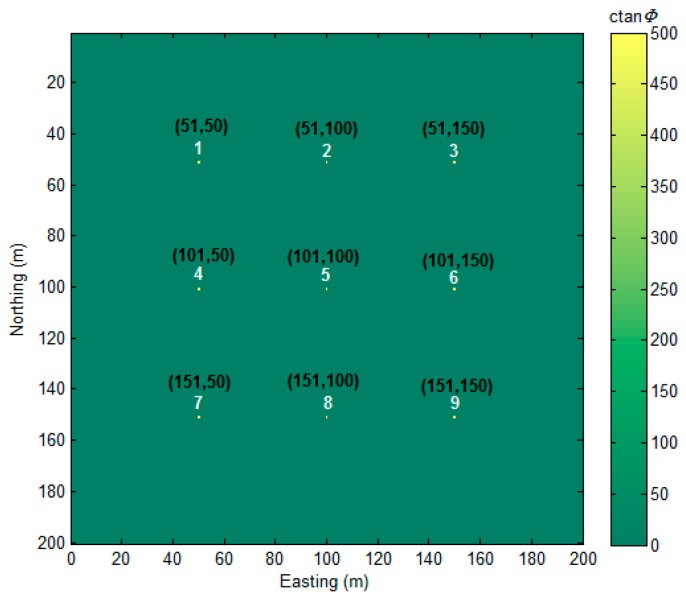
Magnetic Dipole Tangent Angle (MDTA) map of magnetic gradient tensor data. Black text represents the coordination of detected magnetic dipole location.

For the reason of the symmetry of tensor components, we only draw five independent tensors ($\Phi_{xx}$, $\Phi_{xy}$, $\Phi_{xz}$, $\Phi_{yy}$, $\Phi_{yz}$) and $\Phi_{zz}$, according to the expression of Equation (2) in Figure 3. As Figure 3 shows, the anomalous caused by the magnetic dipoles decay quickly with the buried depth increasing. The proposed method was applied to this synthetic model, and the sources detected are shown in Figure 4. The numerical range of the MDTA map is 0–10,853. For visibility, we limited the maximum value to 500, and the threshold parameter α was set to 0.

**Figure 5:**
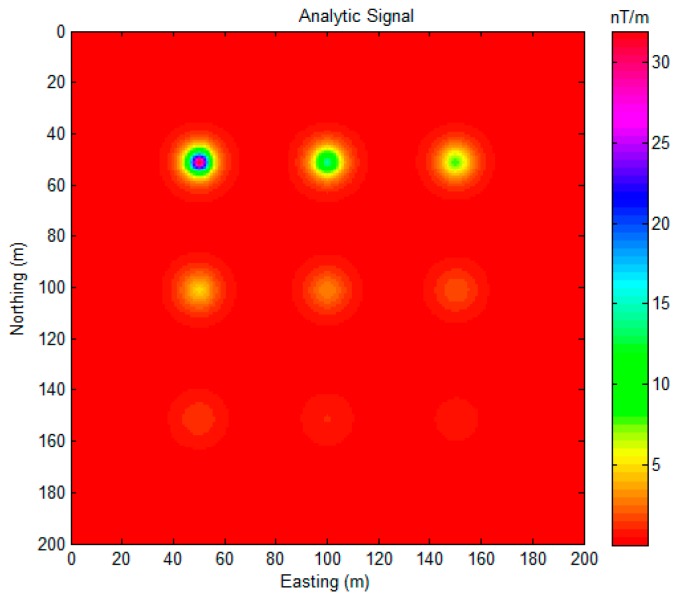
Total field anomalies of the synthetic model.

All of the locations of the magnetic dipole sources were correctly found, as shown in Figure 4. The 1 m estimation error in the east direction, is likely caused by the data sample procedure. Analytic signal (AS) is a useful method in magnetic data interpretation [28]. To compare to it, we drew an AS map (Figure 5). 

As Figure 5 shows, the position of the shallow magnetic dipole sources (1–5) can be located, but the deep magnetic dipole source was not clearly detected with the AS map for this application, as an obvious peak value did not appear for the deep source regions. 

**Figure 6:**
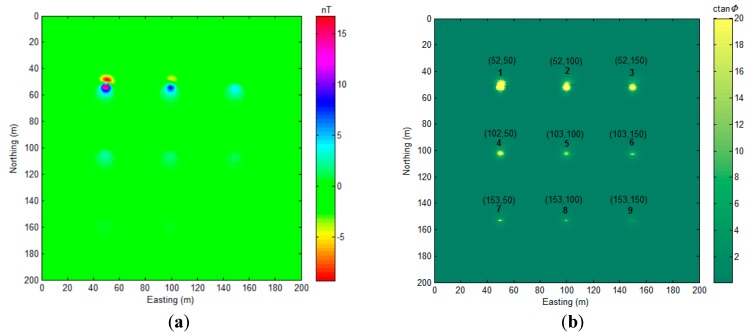
Total field anomaly (**a**) ($I=40^{\circ }，D=10^{\circ }$) and MDTA map (**b**). Black text in (**b**) represents the coordination of detected magnetic dipole locations, α=0.

Then, we changed the declination and inclination of this model (Figure 2) to $I=40^{\circ }，D=10^{\circ }$. The total field anomaly and the MDTA map are drawn in Figure 6. 

**Figure 7:**
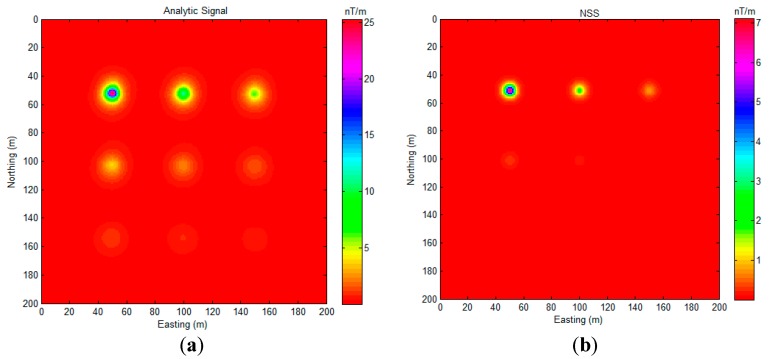
(**a**) Analytic signal (ASS); and, (**b**) Normalized Source Strength (NSS) [11].

Although Figure 6b shows a much wider source location range than Figure 4 for every dipole location, there exists obvious peak values at the corresponding dipole locations, and we mark these positions in Figure 6b with black text. The numerical range of the MDTA map (Figure 6b) is limited from [0, 355] to [0, 20] for visibility. As Figure 6a shows, with the vertical inclination angle ($I=40^{\circ }$) bias, the magnetic anomaly field displays an asymmetrical numerical distribution, but this does not severely affect the precision of the proposed algorithm. Although the Reduce to Pole (RTP) algorithm can reduce this bias, RTP is beyond the scope of this study, and we do not discuss it any further here. We also compared the proposed algorithm with other UXO detection algorithms with this model ($I=40^{\circ }，D=10^{\circ }$), as shown in Figure 7.

As Figure 7a,b shows, analytic signal and normalized source strength do not perform very well for detecting the deep sources in this experiment. 

**Figure 8:**
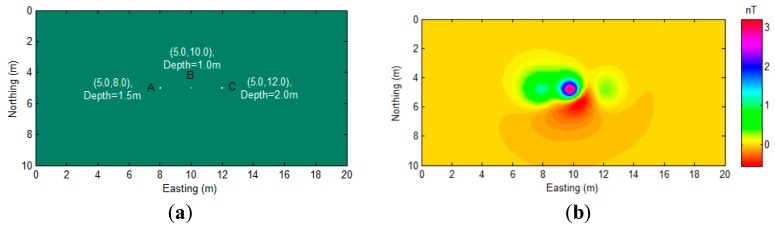
(**a**) Plain sectional view of synthetic model where source A: $I=70^{\circ }，D=30^{\circ }$, and source B: $I=60^{\circ }，D=40^{\circ }$, and source C: $I=80^{\circ }，D=20^{\circ }$; dipole moments are 20 Am^2^; and (**b**) Magnetic anomaly field.

To prove the robustness of the proposed algorithm, we designed a multiple-source scenario with three magnetic dipoles overlapping and close to each other, as shown in Figure 8.

**Figure 9:**
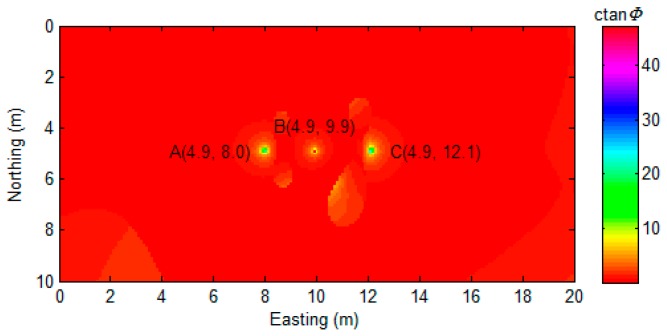
MDTA map. Black text denotes the coordination of detected magnetic dipole locations. (*α* = 0).

Then, the proposed algorithm was applied to the data (Figure 8b), and the result is displayed in Figure 9.

**Figure 10:**
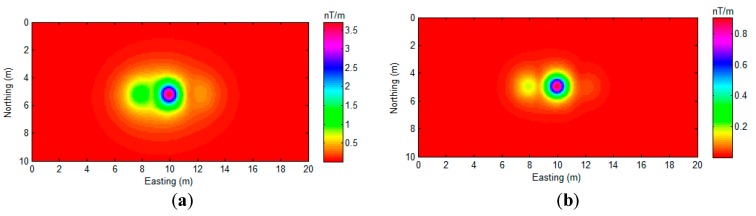
(**a**) AS map of Figure 8 data, and (**b**) NSS map of Figure 8 data.

Although there are some non-zero values shown in the region that are far away from the source position, these values are relatively small and distributed smoothly. It is easy to distinguish them from the large peak values that are related to the actual sources. Overall, the proposed algorithm indicates the position of all of sources with minimal bias, as shown in Figure 9. We also compared the proposed algorithm with AS and NSS, as shown in Figure 10.

**Figure 11:**
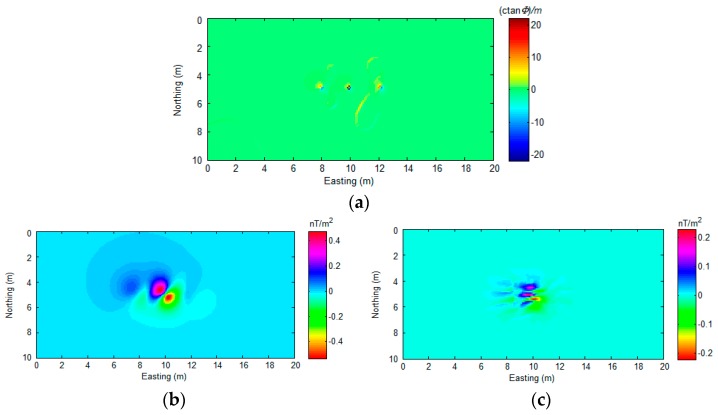
The gradient of the detection results for (**a**) Figure 9, (**b**) Figure 10a, and (**c**) Figure 10b.

As Figure 10 shows, the analytic signal method successfully detects all of the sources (Figure 10a). The NSS method shows more location precision for source A and source B detection. However, both of these two methods could not indicate the exact position of the magnetic dipoles. The numerical distribution in Figure 10a,b is relatively smooth, and it is hard to mark the exact coordinates of the sources. As proof, the gradient of Figure 9 and Figure 10 are calculated and displayed in Figure 11.

Analytic signal and NSS methods perform well in many UXO detection applications. Especially when the observation point is close to the sources, the range of the anomalous sources will be relatively small, and the sources easy to locate. However, when the observation point is far from the UXO, such as during high-altitude air-borne surveys or with deeply buried UXO, it is necessary to develop a corresponding method to locate the source position precisely. Moreover, we believe that locating the source’s horizontal position with a high precision may also increase the computational accuracy in a source depth estimate.

## 4. Study Region and Field Data

**Figure 12:**
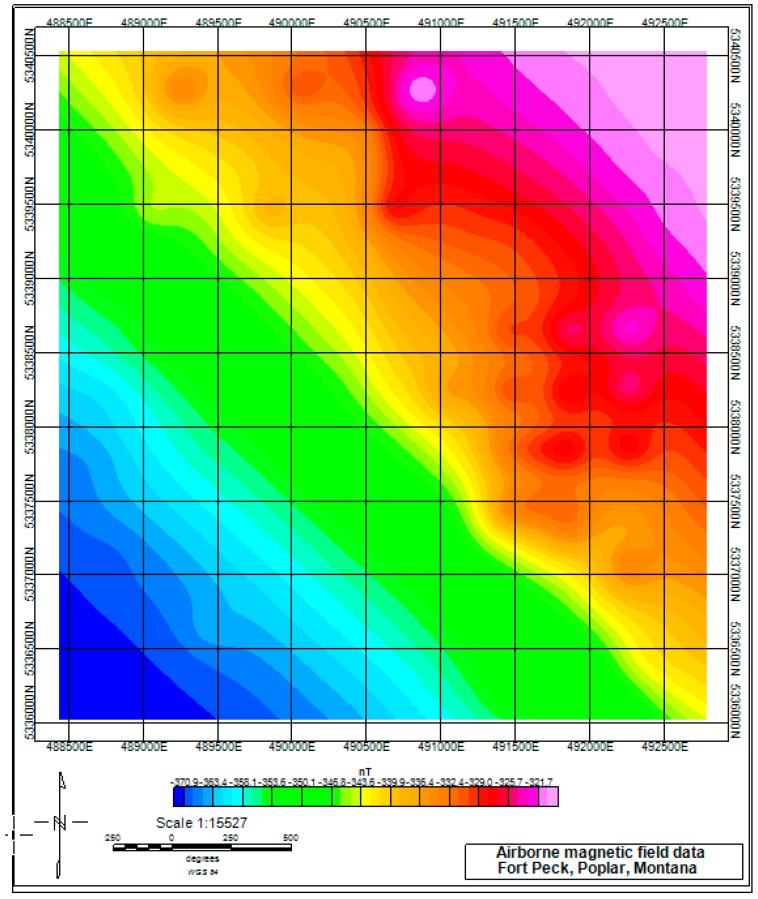
Magnetic field data of part of the East Poplar oil field.

We applied the proposed algorithm to the magnetic field data that was acquired in eastern Montana. This helicopter magnetic survey was conducted by the U.S. Geological Survey (USGS), which includes the East Poplar oil field on the Fort Peck Indian Reservation to assess the ground-water quality. The magnetic data were acquired using the Geometrics G-822 magnetometer, which was mounted on the SkyTEM 301 airborne electromagnetic system. The raw magnetic data were corrected for the diurnal drift, removing the International Geomagnetic Reference Field (IGRF). The reduced-to-pole (RTP) magnetic field was calculated according to the inclination and the declination parameters that were provided in the raw file (Figure 12).

**Figure 13:**
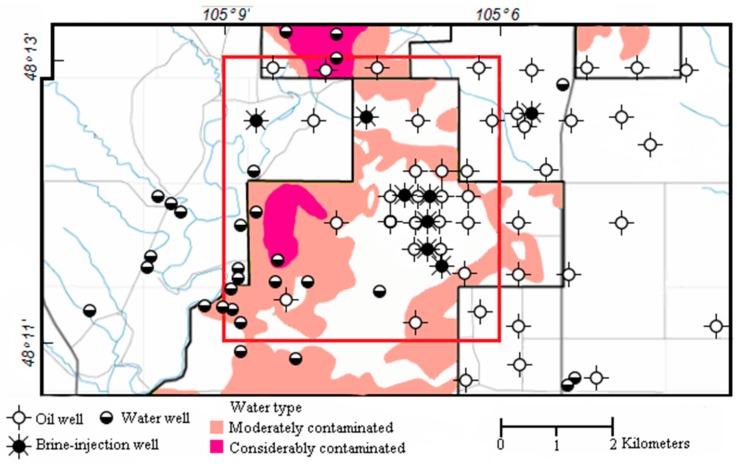
The location of the wells in the research region marked by red rectangle.

The flight altitude was 850 m, and the observation distance was much larger than the size of the metallic oil and water/brine wells, so that the wells can be considered as the magnetic dipoles in this application. The anomalous caused by these dipole-like sources are weak. While the locations of the well targets have been well marked with exact ground prior-information, as shown in Figure 13.

The survey area lies in the Williston Basin, which is a large structural basin with an area of 800 × 500 km. The sedimentation of the Williston Basin formed mostly in the Paleozoic and early Mesozoic eras, and ceased by the Cretaceous era. The thickness of the entire sedimentary section is about three kilometers. Although there is a subsidiary domal structure in the study area, named the Poplar Dome, the geological framework of the study area in the East Poplar oil field is relatively clear and simple [29].

## 5. Results

**Figure 14:**
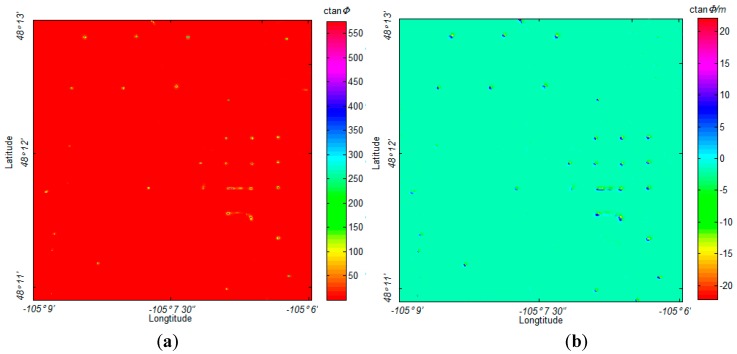
(**a**) *MDTA* map of the field data (*α* = 20); (**b**) Horizontal gradient map of (**a**).

The corresponding gradient tensor maps of this data were calculated by the formulae suggested by Pedersen and Rasmussen [27]. The MDTA map of the field data and the corresponding horizontal gradient map are displayed in Figure 14. 

**Figure 15:**
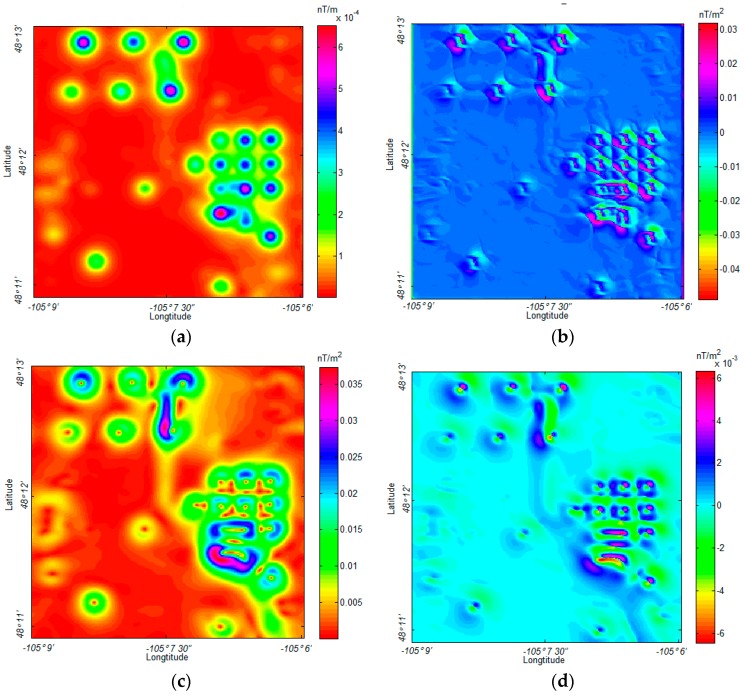
(**a**) NNS of the field data; (**b**) horizontal gradient of (**a**); (**c**) analytic signal of the field data; and, (**d**) horizontal gradient of (**c**).

This magnetic field data was also analyzed with other UXO detection algorithms, as shown in Figure 15.

**Figure 16:**
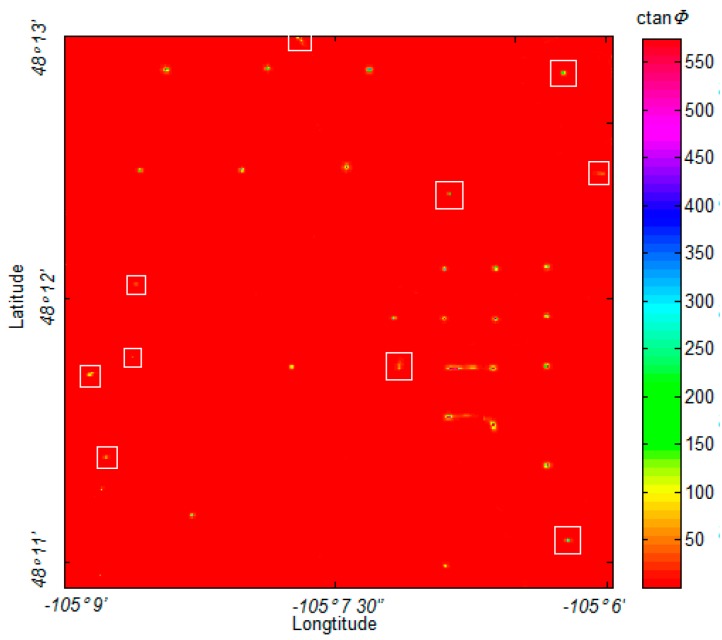
MDTA map marked with the detected weak anomalous sources, identified with a white rectangle.

Both the NSS and analytic signal methods successfully located the primary oil wells in the range, as shown in Figure 15. However, as displayed in Figure 15a, the NSS method performs poorly with weak sources detection in this application, showing some confusion when identifying and describing the weak anomalous source. Figure 15b shows that the weak anomalous sources do not have an intense response on the gradient map. Similarly, the analytic signal shows a relatively precise location for the primary oil wells, as shown in Figure 15c,d, but there is severe interference when the sources are close to each other. In this application, the proposed MDTA was not influenced by these problems, while it marked almost all of the weak anomalous sources and separated the sources located in close proximity to one another. To clearly show this, we marked these weak anomalous sources that were detected by MDTA in Figure 16.

This data contains high-level noise, and it was further amplified in the calculation of tensor components. However, this experiment shows that MDTA is not obviously influenced by the noise and error. Actually, we believe that real field data acquired by a full tensor magnetic gradiometry system will provide better results with the proposed algorithm. 

## 6. Discussion

A common problem with air-borne magnetic surveys is that the anomalies are weak when the distance between targets and observation point is large. The anomalies produced by a magnetic dipole-like source decay with distance cubed inversely. Like the field data in this paper, the amplitude of a weak anomaly may be confused with observation noise and cannot be directly extracted with traditional detection methods. As analyzed in Figure 1, a magnetic dipole can display an obvious direction information in the eigenvector. While the noise contained in the data will not show similar physical properties in eigenvector decomposition. So, the proposed algorithm extracted more of the weak anomalous sources efficiently in the field data experiment. The core of the proposed method is rotating the coordination of the observation plane, and we used the new coordination system to locate the position of magnetic dipoles. Basically, the proposed method cannot be classified as a mathematic transformation, such as Independent Component Analysis (ICA), or Principal Component Analysis (PCA). 

There were some reasons for selecting this magnetic survey data in the field experiment section. Firstly, the location of the metallic objects, such as the oil wells and water/brine oils, were well known prior to this study. This information was critical for validating the proposed algorithm in the field data experiment. Secondly, the altitude of this magnetic survey is high, at 850 m, so the data contains some weak anomalous sources that can be used to prove the efficiency of the proposed algorithm. Although the detected sources are not totally buried under the surface, the covered layer do not have an important influence on the air-borne magnetic survey for the detection of the metallic objects.

Many methods have been developed to detect metallic targets, most of which consider these objects as magnetic dipoles. Commonly, a magnetic dipole has three degrees of freedom related to the source location, while another three degrees are present in the direction and magnitude of the magnetic dipole. In practice, the uncertainty of the direction of the magnetic moment significantly complicates UXO detection. Unlike the remnant magnetization, it is impossible to estimate the direction of every dipole. So, many methods have been proposed to extract the amplitude of the magnetic gradient data, such as the analytic signal and NNS, which are used independently of the magnetization direction. However, these methods are designed to limit the influence of remanent magnetization in an inversion procedure. They build an envelope of magnetic data for all moment directions. Although the uncertainty of magnetization direction can be removed with these transforms, the positioning information of the sources also will be reduced. In UXO detection applications, the goal of the algorithm is to extract the source location parameters, and the direction of the magnetic dipoles is not our main concern. This study analyzed the physical procedure of tensor eigenvector decomposition and directly calculated the horizontal position of a magnetic dipole source. Compared to the analytic signal and NNS methods, the proposed method performs with higher precision in locating the source, and is not severely influenced by the direction of magnetic moment. 

## 7. Conclusions

In this paper, an attempt to locate metallic objects using eigenvector analysis for full tensor magnetic gradient data is presented. The physical meaning of the magnetic gradient tensor data decomposition has been expressed in detail. We used the characteristics of tensor data decomposition to locate the dipole-like magnetic source. The synthetic experiments show that the proposed method is not severely interfered with by the direction of the magnetic moment, and it can be used as an efficient tool to locate weak anomalous sources. We also validated that the method works well when the sources are close or overlapping each other in the synthetic experiment. In practice, the proposed algorithm can be used for both the total-field-magnetic data and magnetic gradient tensor data, and can also help provide information on the horizontal position of the source in modelling or inversion. Future improvements to this work should consider estimating the depth parameter of sources for UXO data interpretation.
